# Detecting motor symptom fluctuations in Parkinson’s disease with generative adversarial networks

**DOI:** 10.1038/s41746-022-00674-x

**Published:** 2022-09-09

**Authors:** Vishwajith Ramesh, Erhan Bilal

**Affiliations:** 1 Department of Biomedical Informatics, University of California, San Diego, CA USA; 2 T.J. Watson Research Center, IBM Research, Yorktown Heights, NY USA

**Keywords:** Parkinson's disease, Machine learning

## Abstract

Parkinson’s disease is a neurodegenerative disorder characterized by several motor symptoms that develop gradually: tremor, bradykinesia, limb rigidity, and gait and balance problems. While there is no cure, levodopa therapy has been shown to mitigate symptoms. A patient on levodopa experiences cycles in the severity of their symptoms, characterized by an ON state—when the drug is active—and an OFF state—when symptoms worsen as the drug wears off. The longitudinal progression of the disease is monitored using episodic assessments performed by trained physicians in the clinic, such as the Unified Parkinson’s Disease Rating Scale (UPDRS). Lately, there has been an effort in the field to develop continuous, objective measures of motor symptoms based on wearable sensors and other remote monitoring devices. In this work, we present an effort towards such a solution that uses a single wearable inertial sensor to automatically assess the postural instability and gait disorder (PIGD) of a Parkinson’s disease patient. Sensor data was collected from two independent studies of subjects performing the UPDRS test and then used to train and validate a convolutional neural network model. Given the typical limited size of such studies we also employed the use of generative adversarial networks to improve the performance of deep-learning models that usually require larger amounts of data for training. We show that for a 2-min walk test, our method’s predicted PIGD scores can be used to identify a patient’s ON/OFF states better than a physician evaluated on the same criteria. This result paves the way for more reliable, continuous tracking of Parkinson’s disease symptoms.

## Introduction

The standard for assessing motor symptoms associated with Parkinson’s disease (PD)—tremor, postural instability, gait difficulty, and bradykinesia—is the motor examination section of the Unified Parkinson’s Disease Rating Scale, or UPDRS Part III. This test is conducted in-person by clinicians and has good inter-rater reliability^1^. However, PD is characterized by sporadic symptoms that are often not observed during clinic visits. For example, freezing of gait (FoG), which causes postural imbalance and frequent falling, has been shown to be difficult to elicit during medical exams. More broadly, it is well-established that the range and severity of symptoms experienced by a patient in their home environment do not always agree with measurements taken in clinic^2–4^. Discontinuous monitoring through clinical visits and in-person assessments do not capture PD symptoms and their progression completely. In fact, patients’ self-assessments of their improvement over time in response to treatment do not agree with their UPDRS scores from in-person appointments^5^.

ON/OFF cycles are another defining characteristic of Parkinson’s disease. A patient treated with a dopamine precursor drug like levodopa experiences the ON state when the drug is active and motor symptoms are less severe. As the drug wears off and motor symptoms worsen, the patient transitions to the OFF state^6^. When a patient visits a neurologist at a clinic, they are either ON, OFF, or transitioning into one of the states. The neurologist can identify the patient’s state during a visit using the symptom severity measured with the UPDRS exam. But without continuous monitoring, it is not possible to capture the relevant dynamics—time in each state, the severity of the OFF state, frequency—of these ON/OFF cycles. Cycles are dependent on the progression of PD and are often unique to individual patients. Understanding the dynamics of these cycles is important because they inform clinicians of the effectiveness of their treatment regimen and the degree of rehabilitation of the patient in a personalized way^7–9^.

The standard for tracking ON/OFF cycles more continuously than in-person clinical visits is the Hauser diary, a major endpoint in PD clinical trials^8,10,11^. Patients monitor the severity of their symptoms and report their ON/OFF state in a home diary every 30 min over the span of several days. PD patients can determine their own ON/OFF state well, by correctly perceiving their non-motor and especially their motor function^12^. Despite this, Hauser diaries are often inaccurate and unreliable^8,13,14^. Poor patient compliance, recall bias, and diary fatigue are common, well-established problems with both paper and electronic diaries^8,13,15^. Due to poor adherence by patients and the fact that diaries only measure the duration of time spent in a state and not the severity of impairment, Hauser diaries are a limited source of information about patients’ physical functions at home.

Moreover, individual UPDRS tasks can benefit from a more objective quantification of symptoms. Gait itself is scored on a scale of 0 to 4 from “Normal” to “Severe”. Examiners are asked to consider stride amplitude, stride speed, height of foot lift, heel strike during walking, turning, and arm swing^16^. Assessing all these independently and then combining them into a single score is a very subjective process and involves a good deal of intuition derived from the experience of the examiner.

Wearable sensors address all the above concerns^13^. They have been shown to have high biomechanical resolution for quantitatively assessing gait impairment in PD, so they have a high degree of clinical applicability^3^. They especially benefit from being able to be taken out of clinic and worn continuously at home or “in-the-wild”. In other words, as Parkinson’s disease patients are simply going about their day, their gait metrics, and motor symptoms can be continuously tracked and ON/OFF cycles automatically monitored. It is important to be able to measure PD outcomes objectively without bias, reliably, and in an unobtrusive way. In-the-wild tracking of the progression of PD in patients via wearables increases the relevance of any clinical visits and improves overall patient management by clinicians^3,13^.

There have been several successful attempts at tracking PD symptoms using wearable sensors and machine learning. Sama et al. were able to detect and score bradykinesia, the slowness of movement, using a waist-worn accelerometer and a support vector regression model^17^. By using machine learning to characterize the walks and strides of patients, they were able to detect and score bradykinesia in 12 subjects with high accuracy and correlation. However, the work used a small subject pool of only 12. And while they were able to achieve high accuracies, the authors used a leave-one-subject-out methodology in which each subject was used to not only train the model but also test it once^17^. The model, therefore, was trained and tested on signals from the same set. Without out-of-sample testing where the entire data from a subject is set aside, models that use few subjects are prone to overfitting.

Another similar study by Rodriguez-Martin et al. used support vector machines (SVMs) to detect FoG events from a single waist-worn sensor at home, to address the lack of FoG events that occur during clinical visits^2–4^. The study’s subject pool was larger at 21. Their model also used a leave-one-subject-out approach but did not perform as well as current standards for detecting FoG. And while their alternative, patient-specific model outperformed the standard, it was personalized in such a way that it involved training on 50% of a patient’s data and testing on the other 50%. While more nuanced, this personalized model was simply a modified leave-one-subject-out approach that used data from 20 subjects and half of the sensor data from 1 test subject to train the algorithm, with the remaining half used to test the model^4^. The model was therefore trained and tested on the same (test) subject’s data.

More recent work also used similar approaches. Studies by Rastegeri et al., Rovini et al., and Chomiak et al. tested several common machine learning algorithms for sensor-based gait analysis and diagnosis of PD, including SVMs, random forest, and naive Bayes^18–20^. All three studies used a cross-validation strategy to train their models (fivefold, tenfold, and Monte Carlo cross-validation, respectively) and reported high performance (accuracy >95%). As in the studies by Sama et al. and Rodriguez-Martin et al., this training paradigm enabled the authors to make the most use of the small sample data that they had collected—10 healthy controls and 10 PD subjects in ref. ^19^, 30 healthy and 30 PD in ref. ^20^, and 9 controls and 21 PD in refs. ^4^^,^^17–20^. Note, however, that while models trained via cross-validation have lower bias, they also tend to have high variance, giving unreliable estimates particularly for classification involving multiple classes^21,22^. Furthermore, notably with leave-one-out, removing one or more examples corresponding to the minority label as part of training will encourage the model to predict the majority class^22^. This is of high concern with healthcare datasets as they tend to be imbalanced; either there are more healthy than sick subjects or subject recruitment in clinics favors sick subjects since healthy individuals do not often go to a hospital. The accuracy values reported in these studies are therefore merely upper bounds on real-world performance. These inconsistencies motivate the use of a more rigorous training paradigm—one that uses a training set, an optional development set or a validation set for tuning hyperparameters, and a separate, independently collected test set that the classifier has never seen. This more rigorous paradigm is a better indicator of the generalizability of a trained model. However, the small size of healthcare datasets and their class imbalance remain problematic—there is not enough data to split into multiple sets for training, validating, and testing of models.

Despite their shortcomings, the studies demonstrated the ease with which signals can be acquired using wearable sensors. And while not ideal, the leave-one subject-out methodology did seem to demonstrate a connection between the time-series accelerometer signals captured during scripted events like walking and PD symptoms like bradykinesia or FoG.

The benefit of using SVMs—the machine learning technique used in the studies in refs. ^4^ and ^17^—is that they have relatively few parameters to train^4,17^. Complex deep neural networks have numerous weight and bias parameters to update and hyperparameters (number of layers, neurons per layer, filters, etc.) to tune. The challenge with using deep nets for small datasets like the ones in the previously mentioned studies is that it is easy for models to overfit^23–26^. Such models perform well on the data used to train them but not on unseen out-of-sample test data; they have poor generalizability. Overfitting is exacerbated by the high sampling rate of wearable sensors, which greatly increases the dimensionality of features—the so-called “curse of dimensionality”^27,28^.

Even datasets that are considered “small” for deep-learning standards like the MNIST handwritten digit database and the CIFAR image dataset have at least several tens of thousands of examples each for training and testing^29,30^. By comparison, the Sama et al. study had 12 subjects and Rodriguez-Martin et al. 21^4,17^. Unfortunately, in healthcare, it is challenging to acquire large samples of subjects on the order of thousands of patients or greater. This is due to difficulties associated with patient recruitment and with logistics of data acquisition, which can be involved for both clinicians and patients^31–34^.

Despite small sample sizes, deep-learning applications in PD have shown promise. Camps et al. used an 8-layer one-dimensional (1D) convolutional neural network (CNN) trained on inertial signals from 21 PD patients to detect FoG events^2^. They were able to obtain 90% geometric mean between specificity and sensitivity, outperforming other state-of-the-art wearable-based methods that used SVMs by 7–18%^2,35,36^. To avoid the problem of overfitting, Camps et al. used a data augmentation strategy to stochastically quadruple their training dataset size^2^. Their strategy involved shifting and rotating the windowed time-series inertial signals for a subset of the samples in their dataset; they then added the transformed subset to the training dataset. Because of the stochastic nature of this strategy, the modified instances were different for each training epoch, adding noise to the training process and preventing their CNN model from overfitting.

In line with the concept of data augmentation, we propose to use generative adversarial networks (GANs) to create realistically generated artificial or “fake” samples that can be used with real samples during training. GANs involve the use of two neural networks—a generator and a discriminator—that play an adversarial minimax game. Fundamentally, a discriminator network is trained to output whether a sample is real or fake to minimize its loss function (like traditional CNNs). Concurrently, a generator is trained to create fake samples that “fool” the discriminator and maximize the discriminator’s loss^37,38^. We incorporate PD symptom assessment (a regression model) into this adversarial game to use deep learning on a dataset with a relatively small subject pool while reducing overfitting.

Other studies have shown this to be the case. Odena, A. experimented with a similar type of GAN as the one we propose to use here^39^. A semi-supervised GAN or “SGAN” was designed to learn not only a generative model but also a classifier simultaneously. On MNIST data, Odena, A. was able to show that the classifier component of the SGAN had better classification accuracy on restricted datasets than a regular CNN. In fact, even with as little as 25 training examples, the SGAN outperformed the CNN^39^. The poor performance of the CNN can be attributed to greater overfitting on the small training sets compared to the GAN. Therefore, we believe that GANs will enable us to obtain better performance on smaller datasets with deep neural networks. GAN-based data augmentation strategies have also shown promise in healthcare-focused classification tasks. Synthetic or “fake” examples used in conjunction with real examples from both small and large training datasets have resulted in increased test performance, as shown by Frid-Adar et al. for computed tomography scans of 182 liver lesions, Ratner et al. for a dataset of 1506 mammograms, and Golany et al. for 109,492 electrocardiograms^40–42^.

In this work, inspired by the prior reasoning, we employ adversarial training to develop a neural network-based regression model that can predict the postural instability and gait disorder score (PIGD) of a Parkinson’s disease subject wearing a lumbar inertial sensor. The proposed models are evaluated on their ability to predict PIGD score as well as to accurately classify ON/OFF states from inferred PIGD scores (i.e., predicted PIGD scores should be greater for the OFF state than for the ON state). We use a modified loss function that considers ON/OFF states of subjects to encourage the network to learn meaningful features that are relevant to Parkinson’s disease state, instead of simply learning the difference between various subjects. The models were tested rigorously with a dataset collected independently and at a different clinic from the one used for training. We show that adversarial training of a GAN leads to better performance compared to typical training of a CNN, and that our GAN model outperforms clinicians when determining ON/OFF state from PIGD scores.

## Results

### Description of datasets

We use data collected from PD patients at two different sites: Tufts University and Spaulding Rehabilitation Hospital. In both sites, subjects were recorded while they performed the different tasks required for the UPDRS test under the supervision of a trained clinician. Each task was scored on a scale from 0 to 4, and then added to create the total UPDRS score. The PIGD sub-score was calculated by adding only the scores relevant to posture and gait: arising from chair, gait, freezing of gait, postural stability, and posture.

Subjects were outfitted with APDM Opal inertial sensors strapped around the limbs and torso with stretchable bands^43^. The APDM sensors recorded accelerometer, gyroscope, and magnetometer data at 128 Hz over time, as subjects underwent the UPDRS test.

We briefly describe the two studies—Study 1 and Study 2—below. For more details about data acquisition, consult Erb et al.^13,44^.

1. Study 1—The subjects in this study were recruited at Tufts University. In all, 35 subjects were recorded over 2 visits, one visit when they were in the ON state and one when they were in the OFF state. In each state/visit, they performed the full battery of UPDRS Part III tests, and their sensor data and UPDRS scores were recorded. As per protocol, the UPDRS Part III was timed to take place either immediately prior to a subject’s next dose of levodopa or immediately after the next dose, with self-reported confirmation that the subject was feeling OFF or ON, respectively. When reporting a state, subjects assessed the severity of their non-motor and motor functions to determine whether they felt the re-emergence of symptoms associated with the wearing off of levodopa.
  Motivated by studies that showed the feasibility of remotely assessing PD symptoms with video conferencing software, we recruited two clinicians to score the symptoms of Study 1 subjects from video^45^. We compared the PIGD scores from each of these video raters to those of the live rater (the clinician who conducted the UPDRS Part III exam in-person) to calculate two coefficients of determination or *R*^2^.
2. Study 2—The subjects in this study were recruited at Spaulding Rehabilitation Hospital. A total of 26 subjects were recorded, but 3 were omitted due to missing UPDRS clinician scores. Each of the 23 subjects remaining was recorded up to five times over 6 h, the approximate duration of a full ON/OFF cycle. Consequently, subjects could have been ON, OFF, or somewhere in between—“TRANSITIONING TO ON” or “TRANSITIONING TO OFF”—in each recording/visit. Furthermore, not all subjects completed a full cycle. For example, there were subjects who were recorded only when they were ON or transitioning into the ON state. Other subjects had at least one visit out of (up to) five when they were ON and at least one recording when they were OFF. In each recording, the subjects underwent the UPDRS Part III test and their sensor data and UPDRS scores were collected. The sensor setup used was the same as in Study 1.

**Fig. 1:**
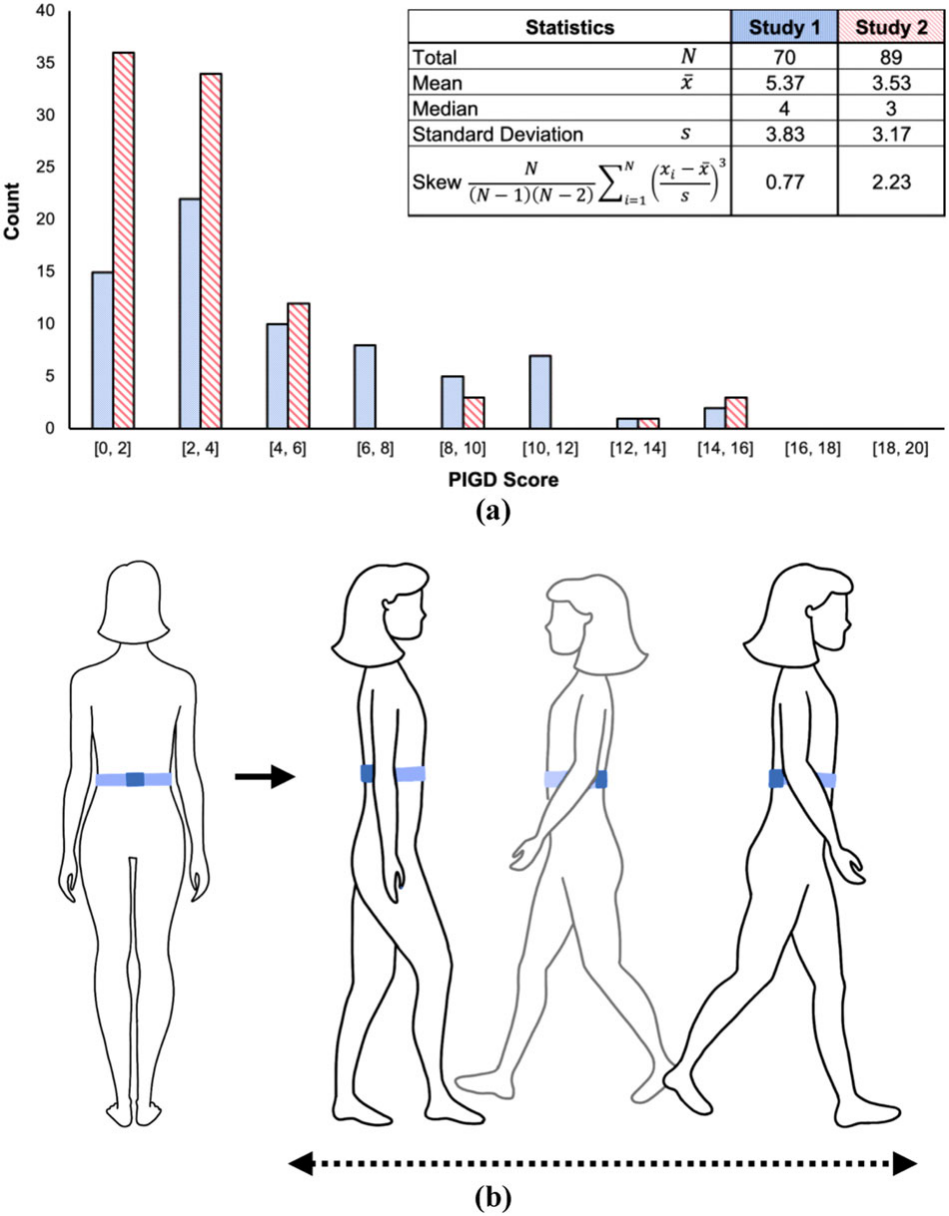
Collecting sensor data from Parkinson’s disease patients. **a** Distributions of PIGD scores for study visits. Both distributions favored lower PIGD scores; distribution for Study 1 is skewed less than for Study 2. **b** Position of an APDM Opal inertial sensor attached to the lumbar region using a stretchable belt. The sensor was placed on the lower back of the subject. Accelerometer, gyroscope, and magnetometer data were collected as subjects walked back and forth for 2 min (as part of the UPDRS Part III test) while wearing this sensor.

There were 70 visits (35 subjects, 2 visits each) for Study 1, each with a self-reported “ON” or “OFF” state. For Study 2, there were 89 recordings with an “ON,” “OFF”, “TRANSITIONING TO ON”, or “TRANSITIONING TO OFF” self-reported state, corresponding to 23 subjects with up to 5 visits each. Figure 1 shows the distribution of PIGD scores for each study, along with a table of statistics, including mean and skew.

We used as features the log spectra of the 3 *X*, *Y*, *Z* acceleration signals from the lumbar APDM sensor that subjects wore as they walked back and forth; we considered only straight walks and not turns. At the end of feature processing (detailed in “Methods”), we obtained 9583 examples total, 4178 examples from the Study 1 dataset and 5405 examples from the Study 2 dataset. Each subject had multiple walks or examples per visit. Each example consisted of 3 spectra (acceleration along the *X*, *Y*, *Z* directions) and was 384 data points long (3 s at 128 Hz, the frequency of data capture of the APDM sensor). The dimensions of the data were therefore 4178 × 3 × 384 for training and 5405 × 3 × 384 for testing. Each example had an associated PIGD sub-score, which was unique to a subject as well as a visit.

We used the larger Study 1 dataset to train our pipelines. Each subject in this dataset had examples from two visits, one visit when they were in the ON state and one when they were in the OFF state. We trained the CNN and GAN with data from 25 out of 35 subjects recorded at Tufts University; 10 subjects were randomly selected for a development set, and their walks left out to be used as a check for convergence. We tested the pipelines using the Study 2 dataset.

Data collection for Study 1 was carried out at the Clinical and Translational Research Center at Tufts Medical Center and all study procedures were approved by the Tufts Health Sciences Campus Institutional Review Board. Study 2 was carried out at Spaulding Rehabilitation Hospital and all procedures were approved by the local Institutional Review Board. Written informed consent was obtained from all participants and all relevant ethical regulations were complied with.

### Training considerations for deep-learning models

We used the larger subject pool recorded at Tufts University to train our deep-learning pipelines, and the Study 2 dataset to test them. While both dataset distributions were concentrated around lower PIGD scores rather than higher scores, Study 1 dataset had more evenly distributed scores. Study 2 dataset had few subject recordings with PIGD scores in the middle; a disproportionate majority of visits had low score labels (below 4) with only a few visits with higher scores (greater than 10). Moreover, skewness was smaller for Study 1 than for Study 2, indicating that the distribution of Study 1 was less asymmetric (Fig. 1). This in particular was important for generalization. A pipeline trained with the more skewed distribution of Study 2 scores would not have performed well when tested with examples not well represented by that dataset, namely walks for subjects with high scores (Supplementary Table 2 confirms the poorer overall performance of models trained on the skewed Study 2 dataset and tested on Study 1).

The networks were trained to take 3-s walks as input and to output a PIGD score. Parameters were updated using the Adam optimization algorithm, an extension to stochastic gradient descent; Adam is a standard for minimizing a parametrized (nonconvex) objective function or “loss” in a computationally effective way^46^. The technique was also used by Salimans et al. in “Improved Techniques for Training GANs”^47^. In this paper, Salimans et al. outlined several methods to encourage loss convergence in the minimax game played by the GAN, an otherwise challenging model to train^47^. One such technique was the historical averaging learning rule, which involved keeping a running average of the parameters of the last few models during training. Any updates that yielded parameters significantly different from this historical average were discouraged (with an L2 cost added to the objective function) to improve convergence. Our implementation of this learning rule was the same as the one in ref. ^47^. While not required, we used historical averaging during the training of the CNN primarily because we used it when training the GAN discriminator. This was done to compare the performance of the two techniques more directly and to better understand the effects of adversarial training. Our deep-learning pipelines were developed using Lasagne and Theano, Python libraries to build and train neural networks and to work with mathematical expressions involving large multi-dimensional arrays^48,49^.

### CNN model and training

CNN performance was the baseline against which we compared the GAN’s performance. To minimize overfitting, the CNN architecture was not deep—two 1D convolutional layers followed by two fully connected layers, with the last fully connected layer also serving as the output of the pipeline. Hyperparameters like batch size and learning rate were empirically determined based on what values yielded the fastest convergence of training losses and the best-performance metrics.

**Fig. 2:**
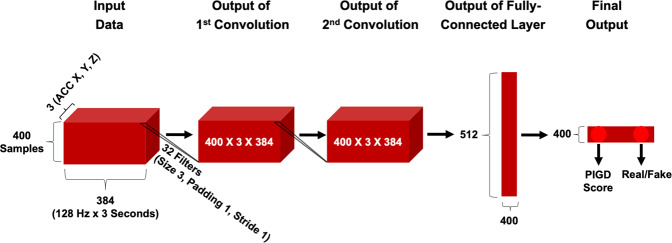
CNN and GAN discriminator architecture. When training the CNN, we disregarded the “Real/Fake” output. Dropout with a probability 0.5 was applied to the output of 2 convolutional and 1 fully connected layer.

We trained with mini-batches containing 400 examples from two visits in Study 1. In the 1D convolutional layers, 32 filters of size 3 operated along the last dimension of the input with stride 1 and padding 1. The 400 × 3 × 384 output of the second convolutional layer was passed to a fully connected layer with 512 units. In order to minimize overfitting, we applied dropout regularization with the probability of 0.5 for all three hidden layers^50^. The last fully connected layer had 2 units for output. Figure 2 summarizes the CNN architecture.

The convolutional layer weights were initialized as an orthogonal matrix^48^. For the two fully connected layers, we used a He initializer with weights sampled from the uniform distribution^51^. All layers except the last output layer used the rectified linear unit (ReLU) activation function. We applied weight normalization to the hidden layers (instead of batch normalization in order to avoid adding noise to an already noise-sensitive regression model)^52^. Biases were initialized as 0’s and the norms of the parametrized weights, *g*, as 1’s.

We used the Study 1 dataset to train the pipeline. Each subject in this dataset had examples from two visits, one visit when they were in the ON state and one when they were in the OFF state. We trained the CNN (and GAN) with data from 25 out of 35 subjects recorded at Tufts University; 10 subjects were randomly selected for a development set and their walks left out to be used as a check for convergence.

In each training step, we randomly selected 200 examples out of 4178. A walk from one of the 25 subjects corresponded to one of the two visits for that subject. If the example came from the first visit, we randomly selected a walk from the second visit for the same subject, and vice versa. Subjects may have appeared multiple times in a mini-batch, but every example from one visit was paired with an example from the opposite visit. We, therefore, obtained mini-batches of size $2 \ast 200 = 400 = N$.

The goal was to learn to differentiate between not only subjects but also the two visits of a subject. Note that the difference in score between subjects was often larger than the difference in scores between the two visits of the same subject. We, therefore, included the mean squared error (MSE) of the difference between two visits in the standard mean squared loss objective function:

1 $$loss\_train = \alpha \ast \frac{1}{N}\mathop {\sum}\limits_{i = 1}^N {\left( {y_i - \hat y_i} \right)^2} + \beta \ast \frac{1}{N}\mathop {\sum}\limits_{i = 1}^N {\left( {\left( {y_{i_1} - y_{i_2}} \right) - \left( {\hat y_{i_1} - \hat y_{i_2}} \right)} \right)^2}$$

2 $$loss\_train = \alpha \ast MSE\left( {y_i,\hat y_i} \right) + \beta \ast MSE\left( {y_{i_1} - y_{i_2},\hat y_{i_1} - \hat y_{i_2}} \right)$$

where *y*_*i*_ is the predicted PIGD score of a subject’s walk, provided by one of the two units in the output layer, and $\hat y$ is the ground truth PIGD score of the example. The first summation term is the MSE between predicted values and ground truth values, weighted by hyperparameter *α*.

In the second summation term, we first calculated the difference between the predicted PIGD score for a walk corresponding to a subject’s first visit $y_{i_1}$, and the predicted PIGD score for a walk from the subject’s second visit $y_{i_2}$. We then calculated this same difference but between the ground truth PIGD values, $\hat y_{i_1} - \hat y_{i_2}$. Lastly, we calculated the mean squared error between these two terms, weighted by hyperparameter *β*. We calculated the second MSE in this way because we wanted to encourage the CNN (and GAN) to learn to make predictions such that the regressed PIGD score of one visit had the same inequality relationship with respect to that of the other visit. That is, if a subject was OFF during their first visit and ON during their second, their PIGD score for the first visit should be higher than that of their second. The predictions made for this example should therefore reflect the same inequality relationship.

In addition to *α* and *β*, other hyperparameters relevant for CNN training included the learning rate and the number of epochs over which we trained. We set *α* and *β* to 1.0 to prioritize differentiating subjects and differentiating visits for a given subject equally during training. The learning rate was fixed at 0.01.

The ten development set subjects recorded at Tufts University that were not used to train the pipeline were used to compute an error at the end of every epoch. We picked these 10 subjects out of the 35 randomly once before training; the 10 subjects remained fixed during training so that we could calculate a loss per epoch and compare it between different epochs. At the end of each epoch, we computed a MSE loss between the predicted and ground truth labels of these ten subjects. We used this development MSE loss as a way of determining the number of epochs over which to train. We trained until this loss converged to a steady-state value (that remained the same for at least 20 epochs). Randomly selecting a different set of ten subjects at the end of every epoch would have made it difficult to reliably compare loss between epochs; it would have been challenging to gauge whether the model was being properly trained if changes in loss could have been due to the subjects randomly chosen for a given epoch.

Supplementary Fig. 1A shows *loss*_*train* (with *α* = *β* = 1.0) plotted over 100 training epochs. We similarly plotted the MSE loss curve computed using 10 Study 1 subjects at the end of every epoch. The fact that both curves converged to some steady-state minimum value confirmed that 100 epochs was enough to complete training,

### GAN model and training

The architecture of the GAN consisted of two neural networks: the generator and the discriminator. The discriminator had the same architecture as the CNN described earlier. This allowed us to directly compare the performance of the CNN and GAN discriminator and to isolate and understand the effects of training with and without data augmentation via a generator network. Henceforth, “GAN discriminator” refers to a shallow neural network trained with an adversarial generator to predict PIGD score and identify real walks from fake ones. “CNN” is the same shallow neural network but trained without a generator that predicts PIGD score alone.

**Fig. 3:**
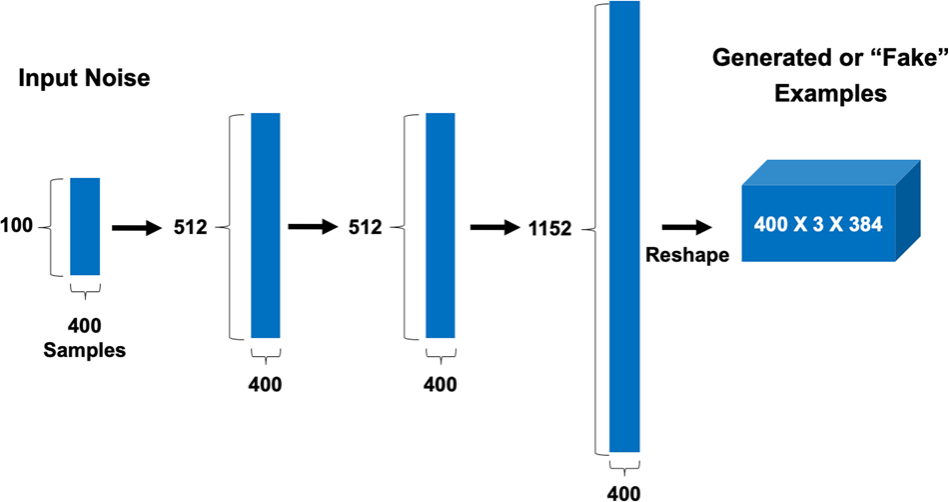
GAN generator architecture. Input noise sampled from the uniform distribution was fed into 3 fully connected layers, and the output of the last was reshaped to the dimensions of the input accepted by the discriminator.

The generator neural network accepted input noise with dimensions 400 × 100, sampled from the uniform distribution between 0 and 1 as in the study in ref. ^47^. The generator architecture consisted of 3 fully connected layers. The first 2 layers had 512 units, used the ReLU activation function and weight normalization^52^. The last layer served as the output layer and consisted of 1152 units with no nonlinearity and L2 regularization. Weights were initialized using the He initializer with the uniform distribution, biases were initialized as 0’s, and the weight norms (*g*) as 1’s^51^. Figure 3 summarizes the GAN generator architecture.

**Fig. 4:**
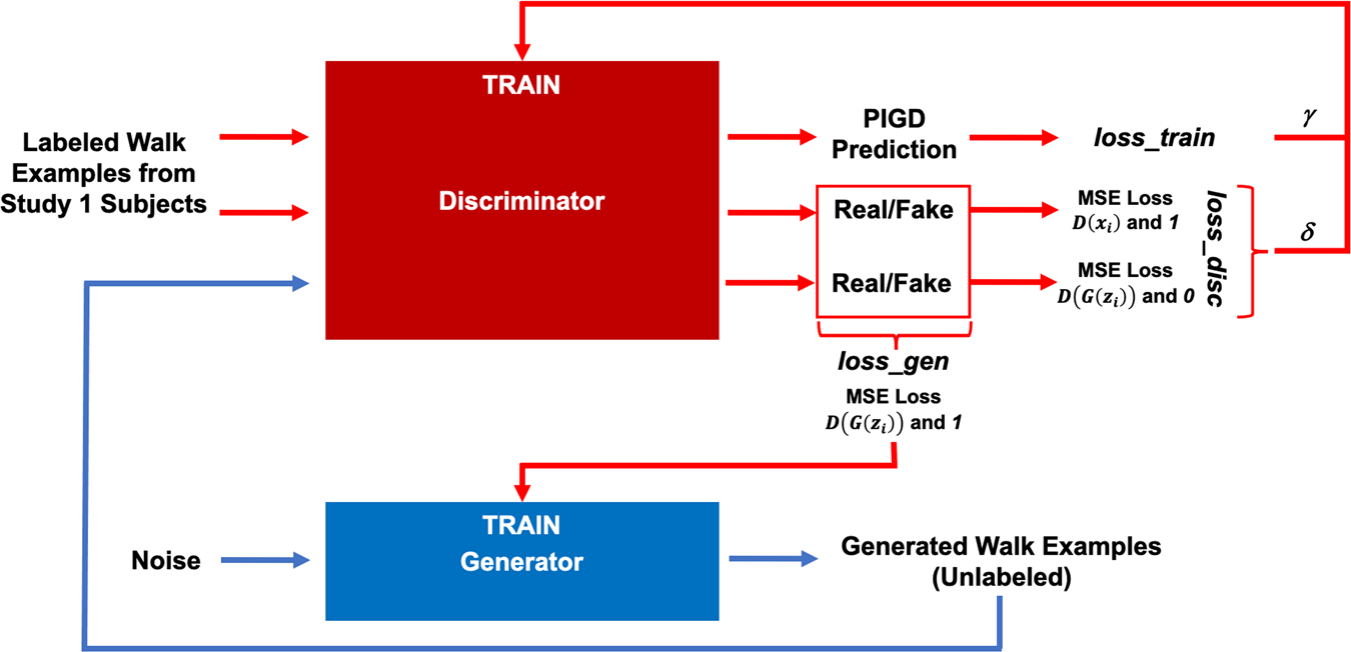
GAN training paradigm. The discriminator predicted a PIGD score as well as a real/fake label for every walk example input. The generator provided fake walk examples. These outputs were used to compute loss terms for training the discriminator and generator. (*loss_train* was also used to train the CNN). See Eqs. (2–4).

In the case of the previous CNN model (trained without an adversarial network) we used only one of two output units in the network; this unit provided the predicted PIGD score. However, when training the GAN, we made use of the second output unit to distinguish real walk examples from fake ones created by the generator. The generator itself was trained to “fool” the discriminator. The training paradigm is detailed below and summarized in Fig. 4.

The discriminator was trained to minimize the following loss, designed to accommodate both PIGD score regression and real/fake example identification:

3 $$loss\_disc = \gamma \ast loss\_train + \delta \ast \left( {\frac{1}{N}\mathop {\sum}\limits_{i = 1}^N {\left( {D\left( {x_i} \right) - 1} \right)^2} + \frac{1}{N}\mathop {\sum}\limits_{i = 1}^N {\left( {D\left( {G\left( {z_i} \right)} \right)} \right)^2} } \right)$$

The function *loss*_*train* was previously defined for training the CNN, except now weighted by the hyperparameter *γ*. $D\left( \cdot \right)$ is the sigmoid output of the discriminator, and identifies whether a sample is real, 1, or fake, 0. Thus, $D\left( {G\left( {z_i} \right)} \right)$ is the discriminator output when a fake walk example $G(z_i)$ is passed in as input. Fake examples were created from noise $z_i$ by the generator $G\left( \cdot \right)$. $D(x_i)$ is the output when a real walk example, *x*_*i*_, is passed in as input. (*i* ranges from 0 to the batch size). The discriminator was essentially trained to push the value of $D(x_i)$ to 1 and $D\left( {G\left( {z_i} \right)} \right)$ to 0.

The generator was trained to minimize the following loss, designed to “fool” the discriminator by pushing $D(G(z_i))$ to 1:

4 $$loss\_gen = \frac{1}{N}\mathop {\sum}\limits_{i = 1}^N {\left( {D\left( {G\left( {z_i} \right)} \right) - 1} \right)^2}$$

The interplay between training the discriminator and generator is outlined in Fig. 4. The training protocol was the same as that of the CNN, with a learning rate fixed at 0.01 and data from ten subjects used as a test for convergence.

Parameters *γ* and *δ* allowed us to control the game between the discriminator and generator. For example, a GAN discriminator trained with $\gamma \,>\, \delta$ would behave similarly to the original CNN; the loss term weighted by *γ* and using unlabeled, fake examples from the generator would not be weighted as highly as *loss*_*train*, which used real examples. In the opposite scenario, with $\delta \,>\, \gamma$, the generator would have a greater than equal influence on the training of the discriminator. In our testing, we used the following combinations of $(\gamma ,\delta )$: (1.0, 1.0), (0.1, 0.5), (1.0, 0.5), (0.5, 1.0), and (0.1, 1.0).

Supplementary Fig. 1B plots *loss*_*disc*, *loss*_*gen*, and the MSE loss curve from ten Study 1 development set subjects over epochs (with $\alpha = \beta = \gamma = \delta = 1.0$). All curves converged to some steady-state minimum value in 100 epochs. Convergence occurred for all combinations of *γ* and *δ* as well.

### Testing models and analyzing their output

We evaluated the CNN and GAN discriminator on their ability to determine ON or OFF state from predicted scores—“ON/OFF accuracy.” For a given subject, the OFF state should have a higher PIGD score than the ON state. The same should hold true for the scores provided by clinicians. After training, we tested the models using the Study 1 development set as well as the Study 2 dataset. Supplementary Table 1 shows ON/OFF accuracy, *R*^2^, and the values of the mean squared terms in *loss*_*train* for the CNN and GAN discriminator for different hyperparameter combinations (we also tested the robustness of the trained models by conducting a parameter sensitivity analysis involving the random perturbation of weights. See Supplementary Fig. 2 and Supplementary Table 3).

Note that each subject had multiple 3-s walks during a recording/visit extracted from the UPDRS gait test. We averaged the predicted PIGD scores of all walk examples for a given visit. We therefore obtained two predicted scores per Study 1 subject, one for each of their visits. For Study 2, we obtained up to 5 predicted scores per subject.

Study 2 subjects could have been ON, OFF, transitioning into the ON state, or transitioning into the OFF state in each of their visits. These labels were self-reported, and it was not a requirement that each subject had visits satisfying all four criteria. Therefore, to simplify our approach, we considered only when subjects were either ON or OFF. We averaged predicted PIGD scores for visits when the subject was ON, *μ*_*ON*_. We similarly averaged the predicted scores for visits when the subject was OFF, *μ*_*OFF*_. Out of 23 subjects, 9 had at least 1 visit when they were ON and at least 1 visit when they were OFF. Of these 9, we counted the number of times $\mu _{OFF} \,>\, \mu _{ON}$. That is, to calculate ON/OFF accuracy, we counted the number of correct ON/OFF determinations made by the CNN or GAN discriminator and divided by total number of examples. We also considered the clinician PIGD scores $\hat \mu$, and assessed whether $\hat \mu _{OFF} \,>\, \hat \mu _{ON}$. Lastly, as for the Study 1 development set, we computed *R*^2^ with 18 predicted (9 *μ*_*ON*_ and *9 μ*_*OFF*_) and 18 ground truth (9 $\hat \mu _{ON}$ and *9* $\hat \mu _{OFF}$) PIGD scores.

Study 1 subjects reported themselves as either ON or OFF in each visit. For the ten development set subjects, we compared the predicted PIGD score for the OFF state with the predicted PIGD score for the ON state; we counted the number of times out of ten that the former was greater than the latter. We repeated this analysis with scores provided by the clinician rater. We gauged clinician performance via both the PIGD sub-score and the overall UPDRS score. In addition to ON/OFF accuracy, we report the coefficient of determination *R*^2^, calculated using 20 predicted PIGD scores from the CNN or GAN discriminator and 20 ground truth PIGD scores from the in-person clinician rater.

We trained the CNN model in Fig. 2 for 100 epochs on 25 subjects from the Study 1 dataset. We used the ten remaining subjects from Study 1 and nine subjects from Study 2 to test whether predicted PIGD scores could be used to correctly determine ON/OFF states given that scores should be greater for the OFF state than the ON state.

The best performing CNN (with hyperparameters $\alpha = 1.0$ and *β* = 0) correctly regressed a PIGD score that was greater for the OFF state than for the ON state in ten occurrences out of ten, yielding an ON/OFF accuracy of 100%, as reported in Table 1. For comparison, the in-person clinician rater had 100% ON/OFF accuracy for the Study 1 development set (Table 1). The CNN’s ON/OFF accuracy for Study 2 subjects was 78%, matching the in-person clinician rater’s accuracy of 78%.

**Table 1:** Performance of CNN, GAN discriminator, and human raters.

Model or human rater	Subjects	*N*	ON/OFF accuracy	*R*^2^
Best CNN	Study 1 Dev Set	10	100%	0.56
Best CNN	Study 2 Test Set	9	78%	0.61
Best GAN	Study 1 Dev Set	10	100%	0.61
Best GAN	Study 2 Test Set	9	100%	0.55
In-Person Clinician Rater (Ground Truth)	Study 1 Dev Set	10	100%	N/A
In-Person Clinician Rater (Ground Truth)	Study 2 Test Set	9	78%	N/A
In-Person Clinician Rater (Ground Truth)	Partial Study 1	25	64%	N/A
Video Rater 1	Partial Study 1	25	68%	0.45
Video Rater 2	Partial Study 1	25	48%	0.37
Video Rater Average	Partial Study 1	25	58%	0.41

We trained a GAN discriminator with the same architecture as the CNN (Fig. 2) alongside the GAN generator in Fig. 3 for 100 epochs on the Study 1 dataset. (A generated sample is shown in Supplementary Fig. 3). Table 1 shows that at best, the GAN discriminator had 100% ON/OFF accuracy on both the Study 1 development set (with $\alpha = \beta = \delta = 1.0,\gamma = 0.5$) and the Study 2 dataset (with $\alpha = \beta = \delta = \gamma = 1.0$). The GAN discriminator outperformed the in-person clinician rater for the Study 2 dataset.

In addition to ON/OFF accuracy, we compared predicted scores from the CNN and GAN discriminator to the scores from the in-person rater to calculate the coefficient of determination *R*^2^. The *R*^2^ values for the CNN and GAN discriminator were similar. For Study 2, for example, CNN *R*^2^ was 0.61, and GAN discriminator *R*^2^ was 0.55 (Table 1).

**Fig. 5:**
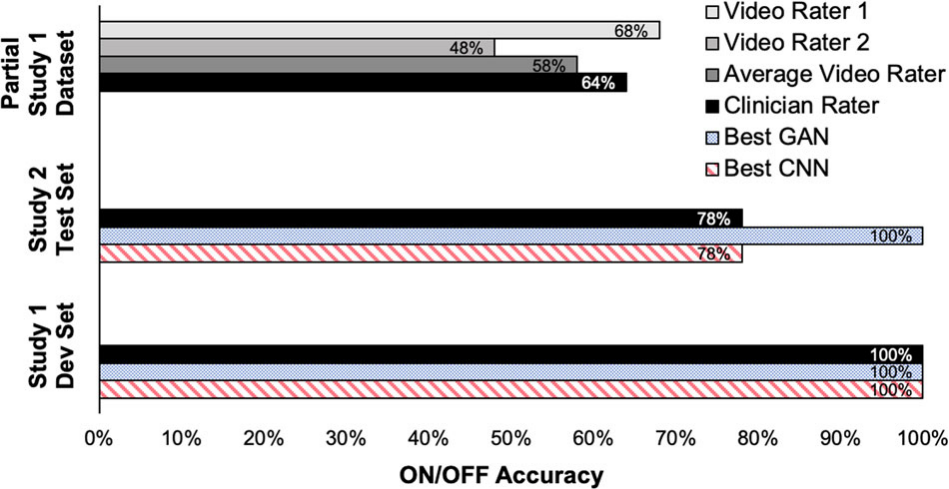
ON/OFF accuracy of deep-learning models and human raters. Only the ON/OFF accuracies for the hyperparameter combinations that yielded the best CNN and GAN discriminator performance are plotted. Models were tested with ten Study 1 development set subjects and nine Study 2 subjects. The ON/OFF accuracies of the in-person clinician rater are also plotted, alongside the performance of two human raters who assessed 25 Study 1 patients over video.

Furthermore, we obtained PIGD scores from two video raters for part of the Study 1 dataset, 25 out of 35 subjects. (These 25 subjects were different from the 25 subjects used to train the models.) We calculated the *R*^2^ values by comparing PIGD scores from each video rater to those of the live clinician and then inferred the accuracy scores as previously described. The performance of the video raters, when considering the PIGD portion of UPDRS, was lower than CNN and GAN models, both in terms of *R*^2^ and ON/OFF accuracy (Table 1 and Fig. 5).

## Discussion

In this paper, we set out to show that we could teach neural networks to predict the PIGD score of a subject with Parkinson’s disease from a single lumbar accelerometer. We used two independent datasets of subjects performing a simple walk test to train, test and validate our approach. Both CNN and GAN models showed performance on par with the in-person clinician rater and outperformed two video raters.

We tested the ability of adversarial training to improve out-of-sample performance of a shallow neural network on the premise that generating synthetic samples from noise can successfully augment real clinical datasets. Our models were evaluated along two dimensions by comparing them to the clinician’s PIGD score and self-reported ON/OFF states. The premise behind inferring ON/OFF accuracy was that PIGD scores that were output from both CNN and GAN models should have been larger for visits when the subject was in the OFF state than visits when the subject was in the ON state. Self-reported ON/OFF labels were used as ground truth because: (1) it has been shown that patients’ perception of their motor functions successfully model PD severity on par with the clinically objective UPDRS exam and (2) self-reported states are often used as a clinical endpoint in PD clinical trials^12^.

GAN discriminator’s ability to predict ON/OFF state was slightly greater than that of the CNN model, at best performing 22% better on the Study 2 subjects. Both models were trained on the same data and the CNN and GAN discriminator had almost identical architectures (Fig. 2). Hence the results suggest that the performance difference was due to the influence of the adversarial generator during discriminator training. Furthermore, the GAN discriminator performed better on out-of-sample Study 2 subjects when the generator had greater than equal influence on discriminator training, i.e., when the second term in *loss_disc* computed using generated examples was weighted more than the first term (when $\delta \,>\, \gamma$, see Supplementary Table 1). We thus hypothesize that generative adversarial training helps counter the curse of dimensionality problem when applying deep-learning techniques on small datasets prevalent in healthcare applications.

When tested on the Study 2 dataset, the best GAN discriminator outperformed a clinician rater when considering their PIGD scores, making no mistakes (Fig. 5). We note though that the clinician rater was ultimately still better when considering the full UPDRS score—88% ON/OFF accuracy instead of the 78% calculated from the PIGD sub-score. However, the clinician had the advantage of assessing symptoms that were not captured by the walk test, such as limb rigidity. We chose to focus on just the walk test because we were interested in developing models that could potentially be taken outside the clinic to automatically monitor patients in-the-wild. Clinical visits are an episodic and discontinuous way to monitor the longitudinal progression of Parkinson’s disease while patient self-report is subjective and adds an additional burden on the patient. We hypothesize that continuous monitoring could be achieved in the future by focusing on UPDRS tasks that are more naturally performed by people in their daily lives, and walking is a prime example of that.

Although ON/OFF accuracies are high, it is interesting to note that the coefficients of determination hover in the 0.50–0.60 range. In other words, the models perform better when comparing them to patient self-reports than when comparing them to the clinician rater. This observation is relatively consistent over multiple hyperparameter choices (e.g., *δ*, *y*, *α, β*, see Supplementary Table 1) so it is unlikely to be a training artifact. A more likely explanation is that there is low inter-rater reliability between clinicians that score UPDRS Part III tasks^53^. The two video raters in this study also show considerable variation in performance when compared to the in-person rater and to each other (Table 1 and Fig. 5).

A drawback of this study is that the models described here were trained on walk sensor data collected in a clinic under a data collection protocol (subjects walked back and forth for 2 min). Walks that occur at home are shorter in both duration and distance, and in-the-wild walks are likely diverse and erratic. The current models may not generalize as well to walks collected outside of a clinic. Further studies should be conducted to collect at-home sensor data. In addition, mode collapse, a growing concern with GANs, was unaddressed in this work but needs to be quantified before deployment^54^. Note, however, that the empirical improvements provided by adversarial training would not have been as large if the generator were producing only a limited range of diverse samples.

We conclude that the training method presented in this work is a promising technique to approach sensor data collected from a relatively small subject pool and the resulting model has the potential to assess postural instability and gait disorder in a way that enables ON/OFF prediction. Going forward, the challenge is to be able to translate this work from the clinic to the home environment. We envision a system that can be used to reliably assess symptoms and track ON/OFF cycles continuously. Doing so will better inform clinicians on the state of the patients and aid in their rehabilitation, ultimately improving their quality-of-life.

## Methods

### Feature processing

As part of the UPDRS Part III, subjects walked for 2 min along a straight line, turned around, returned to the examiner, and repeated this process (Fig. 1). They were outfitted with several sensors, including a lumbar inertial measurement unit (IMU) worn about the torso (Fig. 1). In this work, we considered only this sensor because:

1. It is close to the center of mass of the human body. The trunk is therefore the best sensor location for assessing standing balance, walking stability, and posture identification^55–59^.
2. Wearing a lumbar sensor around the torso towards the front (above the anterior superior iliac spine) has been shown to be more comfortable for subjects^60^.

The lumbar IMU included an accelerometer to measure acceleration in the *X*, *Y*, *Z* directions and a gyroscope to measure rotation speed around the *X*, *Y*, *Z* axes. We therefore obtained six time series as the subjects walked and turned. By using the gyroscope values, we detected and cut out turn events; turns were defined as time periods when the rotation about the *X* axis was larger than 120°. We were therefore left with only snippets of straight walking events. Since subjects walked back and forth, each walk was an example and each subject had several walks or examples per clinic visit. To simplify our approach, we considered only the three acceleration signals.

Some subjects walked faster than others, so we truncated the three IMU acceleration signals at 3 s. Doing so ensured that we maintained the same data dimensions across all subjects. We found that a greater cutoff reduced the number of examples overall, since subjects generally completed a single straight walk in around 3 s. On the other hand, a smaller cutoff resulted in shorter examples with less information, ultimately giving poorer model performance. A 3 s cutoff gave us a good number of examples without encouraging overfitting or sacrificing performance.

The time-series IMU signals were filtered using a high-pass Butterworth filter (0.25 cutoff) to remove drift and gravity effects. Lastly, we converted the time series to log spectra using a Fourier transform. It was natural to look at the Fourier decomposition of the time-series signals because walking is a periodic activity; this periodicity was reflected in the log spectra^61,62^ (examples of the log spectra obtained at the end of this feature processing step are shown in Supplementary Fig. 3).

### Reporting summary

Further information on research design is available in the Nature Research Reporting Summary linked to this article.

## Supplementary information

Supplementary Information

Reporting Summary

## Data Availability

The data that support the findings of this study are available from the two institutions that were the legal sponsors of the studies. Sponsors were responsible for study conduct and data validation/storage. Study 1 was sponsored by Tufts Medical Center and Study 2 by Spaulding Rehabilitation Hospital. Restrictions apply to the availability of these data, which were used with permission for this study, and so are not publicly available. Data are, however, available from the authors upon reasonable request and with permission of the sponsors. Data collection details can be found in ref. ^13^.

## Code availability

We do not have open-source code available. The code for training and testing the deep-learning models were written in Python 2.7 using Theano 1.0.5 and Lasagne 0.2.dev1. Data management and feature processing scripts were written in Python 2.7 and used Pandas 1.1.0 and NumPy 1.18.5. The code used to complete the analysis may be requested by contacting the corresponding author.
