# Supplementary material for: Detecting motor symptom fluctuations in Parkinson’s disease with generative adversarial networks
Source: NPJ Digit Med. 2022 Sep 9;5:138. doi: 10.1038/s41746-022-00674-x (PMC9463161; doi:10.1038/s41746-022-00674-x)
Supplement: Supplementary file 1 — Supplementary Information [file 41746_2022_674_MOESM1_ESM.pdf]

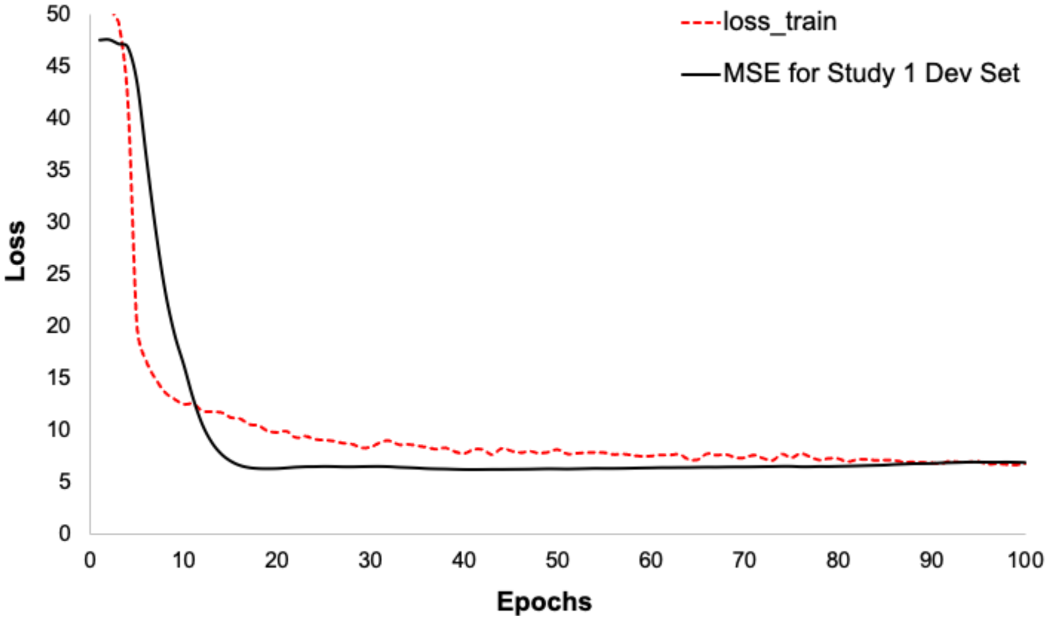

(a)

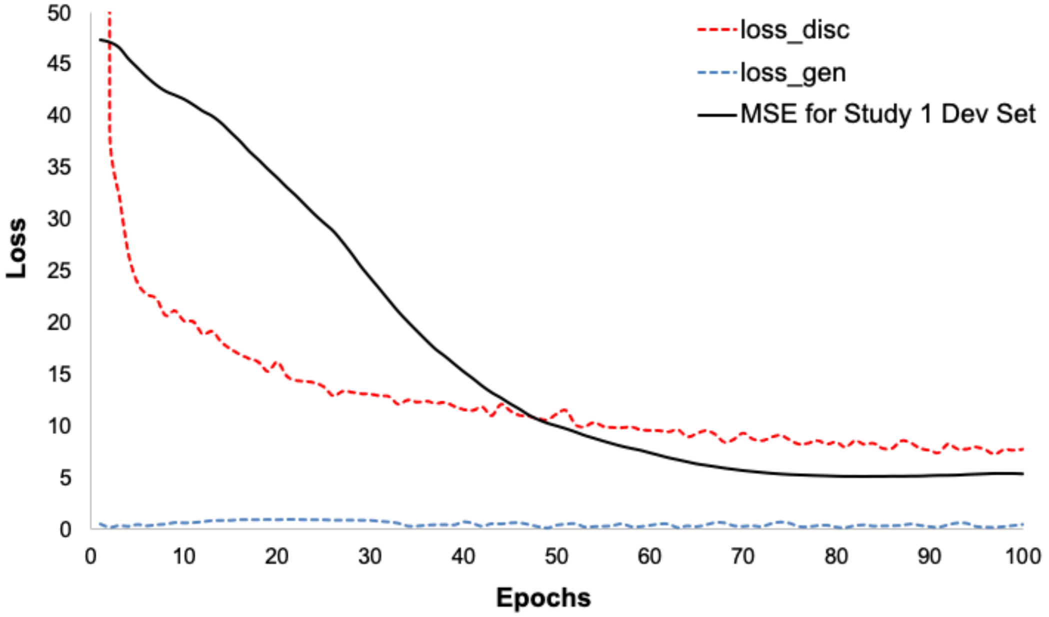

(b)

Supplementary Figure 1. Loss curves. **(a)** Best CNN. *loss_train* is plotted over epochs in gray, and the mean squared error (MSE) loss curve for 10 Study 1 subjects in black. **(b)** Best GAN. *loss_disc* is plotted in red and *loss_gen* in blue over epochs. MSE loss curve for 10 Study 1 subjects is shown in black. All 3 curves are for ($\gamma = 1.0$, $\delta = 1.0$). Note that convergence occurred for the 4 other (γ, δ) combinations tested as well.

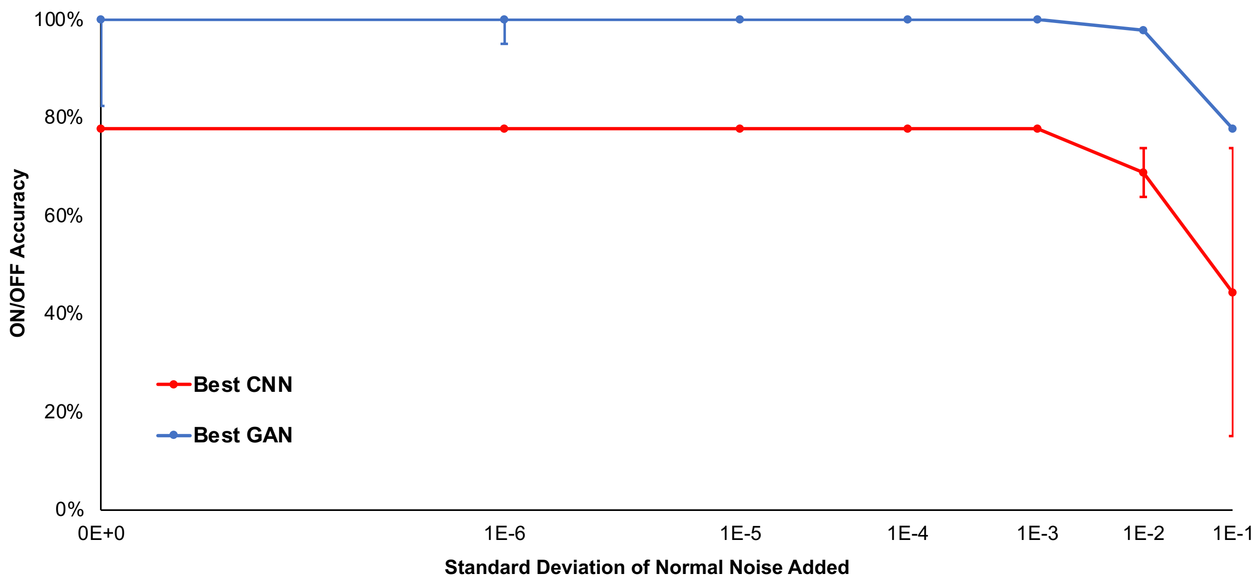

Supplementary Figure 2. ON/OFF accuracy of trained CNN and trained GAN discriminator when different amounts of random normal noise are added to weights. The ON/OFF accuracy values shown were averaged over 5 trials, with standard deviation shown as error bars. Average performance of the GAN discriminator drops from 100% to 98% to 78% (in blue) as more and more noise is added to the weights of the trained model. When random values sampled from the normal distribution with mean 0 and standard deviation 0.1 – the greatest amount of noise – is added to the weights of the trained CNN, we see that ON/OFF accuracy is 44% (in red). These results are overall consistent with our expectation that the accuracy of trained models will decrease when random noise is added to the weights. Both models perform at their peak performance up until a significant amount of noise is added, with a greater drop off in performance for the CNN than for the GAN discriminator.

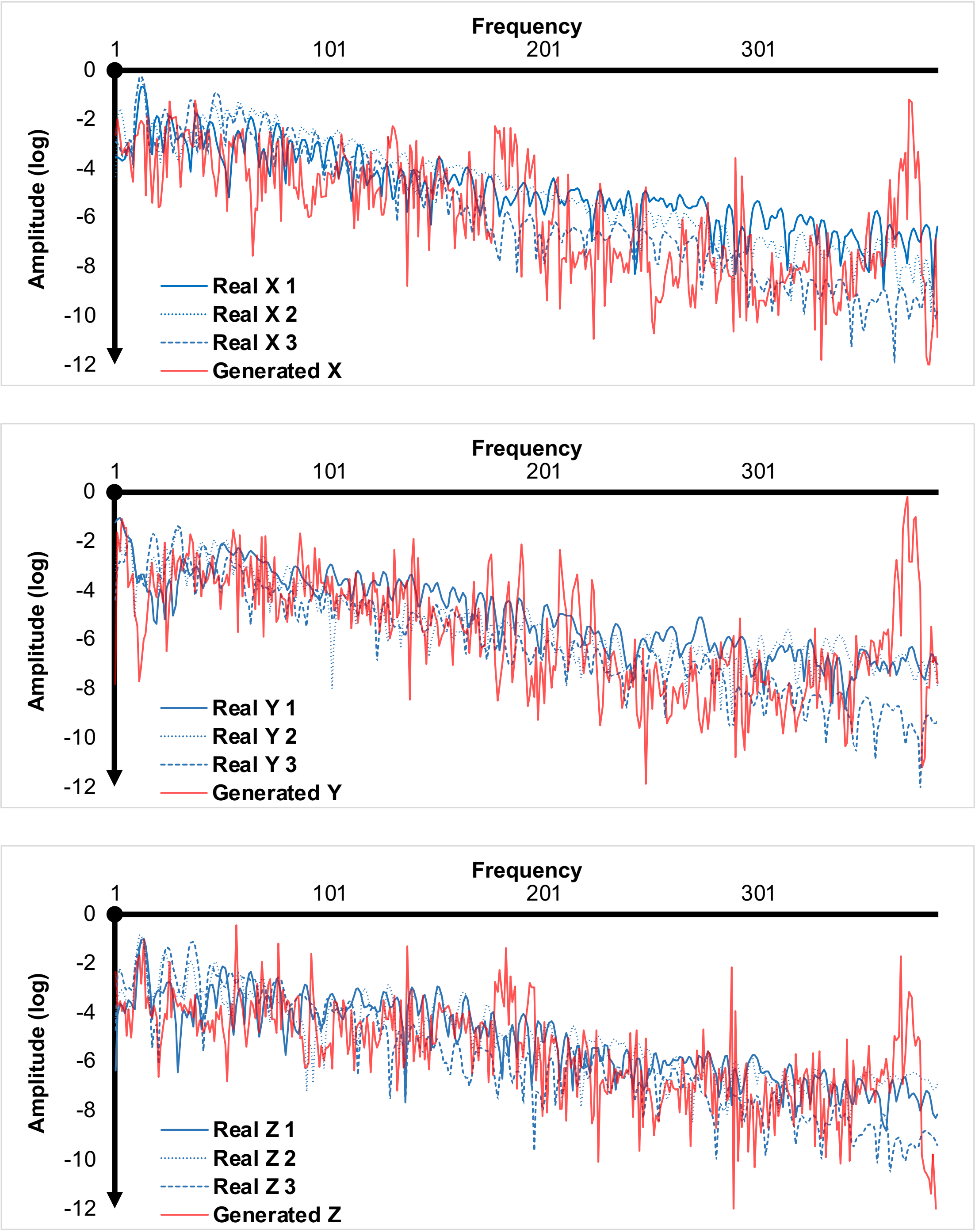

Supplementary Figure 3. Three randomly chosen real samples and one created by the fully trained generator. In blue are the log spectra features - acceleration in X (top), Y (middle), and Z (bottom) - obtained at the end of the feature processing step. In red is a sample created by a generator trained using the real samples.

Model	Subjects	N	Hyperparameters				ON/OFF Accuracy	R2	Losses	
			α	β	γ	δ			$MSE(y_i, \hat{y_i})$	$MSE(y_{i_1} - y_{i_2}, \widehat{y_{i_1}} - \widehat{y_{i_2}})$
CNN	Study 1 Dev Set	10	0.5	1	-	-	80%	0.48	6.35	7.25
			1	0	-	-	100%	0.56	5.74	6.63
			1	0.5	-	-	70%	0.49	7.26	8.69
			1	1	-	-	70%	0.56	5.57	6.26
	Study 2 Test Set	9	0.5	1	-	-	78%	0.37	6.40	3.50
			1	0	-	-	78%	0.56	5.34	2.89
			1	0.5	-	-	78%	0.61	4.48	3.10
			1	1	-	-	67%	0.53	10.73	4.58
GAN	Study 1 Dev Set	10	0.5	1	1	1	70%	0.44	6.61	7.72
			1	0	1	1	90%	0.62	4.36	6.30
			1	0.5	1	1	100%	0.61	4.39	6.03
			1	1	1	1	100%	0.6	4.61	4.21
					0.1	0.5	90%	0.58	4.88	6.06
					1	0.5	90%	0.61	4.34	6.00
					0.5	1	100%	0.61	4.77	5.24
					0.1	1	80%	0.56	5.69	7.00
	Study 2 Test Set	9	0.5	1	1	1	89%	0.32	6.16	4.79
			1	0	1	1	89%	0.47	6.95	5.93
			1	0.5	1	1	89%	0.44	5.23	4.84
			1	1	1	1	100%	0.55	6.75	4.17
					0.1	0.5	89%	0.36	8.62	6.13
					1	0.5	67%	0.54	6.90	4.74
					0.5	1	78%	0.43	10.02	5.28
					0.1	1	78%	0.45	9.85	4.90

Supplementary Figure 4. Performance of CNN and GAN discriminator trained with different hyperparameter combinations.

Model	Subjects	N	Hyperparameters				ON/OFF Accuracy	R2	Losses	
			α	β	γ	δ			$MSE(y_i, \hat{y_i})$	$MSE(y_{i_1} - y_{i_2}, \widehat{y_{i_1}} - \widehat{y_{i_2}})$
CNN	Study 1 Test Set	35	0.5	1	-	-	60%	0.62	12.52	6.76
			**1**	**0**	**-**	**-**	**71%**	**0.51**	**26.54**	**7.93**
			1	0.5	-	-	60%	0.15	14.41	8.08
			1	1	-	-	63%	0.60	6.88	5.82
GAN	Study 1 Test Set	35	0.5	1	1	1	63%	0.40	13.58	6.75
			**1**	**0**	**1**	**1**	**71%**	**0.27**	**27.61**	**7.89**
			1	0.5	1	1	69%	0.37	25.41	7.98
			1	1	1	1	60%	0.47	12.61	6.61
					0.1	0.5	54%	0.30	21.37	7.63
					1	0.5	54%	0.11	26.53	8.02
					0.5	1	69%	0.39	22.37	7.33
					0.1	1	51%	0.39	21.20	7.04

Supplementary Figure 5. Performance of CNN and GAN discriminator trained on the Study 2 dataset and tested on Study 1 data. The hyperparameter combinations for which the models had best ON/OFF accuracy are in bold. As expected, the test performance is lower when the models were trained using the more skewed Study 2 dataset rather than the more balanced Study 1 dataset.

Model	Subjects	N	Hyperparameters				Std. Dev. of Added Normal Noise (Mean 0)	ON/OFF Accuracy*	R2*	Losses*	
			α	β	γ	δ				$MSE(y_i, \hat{y_i})$	$MSE(y_{i_1} - y_{i_2}, \widehat{y_{i_1}} - \widehat{y_{i_2}})$
Best CNN	Study 2 Test Set	9	1	0.5	-	-	0	78%	0.61	4.4800	3.1000
							1.E-06	78%	0.10	16.5729	9.9969
							1.E-05	78%	0.10	16.5727	9.9969
							1.E-04	78%	0.10	16.5761	9.9969
							1.E-03	78%	0.10	16.5837	9.9969
							1.E-02	69%	0.10	16.3223	9.9966
							1.E-01	44%	0.17	16.3835	10.0900
Best GAN	Study 2 Test Set	9	1	1	1	1	0	100%	0.55	6.7500	4.1700
							1.E-06	100%	0.59	3.3592	5.1146
							1.E-05	100%	0.59	3.3592	5.1145
							1.E-04	100%	0.59	3.3589	5.1131
							1.E-03	100%	0.59	3.3601	5.1131
							1.E-02	98%	0.57	3.4324	5.2744
							1.E-01	78%	0.34	8.2301	8.1511

*Averaged over 5 trials

Supplementary Figure 6. Performance of trained CNN and trained GAN discriminator with different amounts of weight perturbation.
