# Supplementary material for: Detecting motor symptom fluctuations in Parkinson’s disease with generative adversarial networks
Source: NPJ Digit Med. 2022 Sep 9;5:138. doi: 10.1038/s41746-022-00674-x (PMC9463161; doi:10.1038/s41746-022-00674-x)
Supplement: Supplementary file 2 — Reporting Summary [file 41746_2022_674_MOESM2_ESM.pdf]

nature portfolio

Corresponding author(s): Vishwajith Ramesh

Last updated by author(s): Aug 2, 2022

Reporting Summary

Nature Portfolio wishes to improve the reproducibility of the work that we publish. This form provides structure for consistency and transparency in reporting. For further information on Nature Portfolio policies, see our Editorial Policies and the Editorial Policy Checklist.

Statistics

For all statistical analyses, confirm that the following items are present in the figure legend, table legend, main text, or Methods section.

n/a	Confirmed	
☐	☒	The exact sample size (*n*) for each experimental group/condition, given as a discrete number and unit of measurement
☐	☒	A statement on whether measurements were taken from distinct samples or whether the same sample was measured repeatedly
☒	☐	The statistical test(s) used AND whether they are one- or two-sided<br>*Only common tests should be described solely by name; describe more complex techniques in the Methods section.*
☒	☐	A description of all covariates tested
☒	☐	A description of any assumptions or corrections, such as tests of normality and adjustment for multiple comparisons
☒	☐	A full description of the statistical parameters including central tendency (e.g. means) or other basic estimates (e.g. regression coefficient) AND variation (e.g. standard deviation) or associated estimates of uncertainty (e.g. confidence intervals)
☒	☐	For null hypothesis testing, the test statistic (e.g. *F*, *t*, *r*) with confidence intervals, effect sizes, degrees of freedom and *P* value noted<br>*Give P values as exact values whenever suitable.*
☒	☐	For Bayesian analysis, information on the choice of priors and Markov chain Monte Carlo settings
☒	☐	For hierarchical and complex designs, identification of the appropriate level for tests and full reporting of outcomes
☒	☐	Estimates of effect sizes (e.g. Cohen's *d*, Pearson's *r*), indicating how they were calculated

Our web collection on statistics for biologists contains articles on many of the points above.

Software and code

Policy information about availability of computer code

Data collection	No software was used by us for data collection.
Data analysis	The code for training and testing the deep learning models were written in Python 2.7 using Theano 1.0.5 and Lasagne 0.2.dev1. Data management and feature processing scripts were written in Python 2.7 and used Pandas 1.1.0 and NumPy 1.18.5.

For manuscripts utilizing custom algorithms or software that are central to the research but not yet described in published literature, software must be made available to editors and reviewers. We strongly encourage code deposition in a community repository (e.g. GitHub). See the Nature Portfolio guidelines for submitting code & software for further information.

Data

Policy information about availability of data

All manuscripts must include a data availability statement. This statement should provide the following information, where applicable:
- Accession codes, unique identifiers, or web links for publicly available datasets
- A description of any restrictions on data availability
- For clinical datasets or third party data, please ensure that the statement adheres to our policy

The data that support the findings of this study are available from the two institutions that were the legal sponsors of the studies. Sponsors were responsible for study conduct and data validation/storage. Study 1 was sponsored by Tufts Medical Center and Study 2 by Spaulding Rehabilitation Hospital. Restrictions apply to the availability of these data, which were used with permission for the current study, and so are not publicly available. Data are however available from the authors

upon reasonable request and with permission of the sponsors. Data collection details can be found in:

Erb, M.K., Karlin, D.R., Ho, B.K. et al. mHealth and wearable technology should replace motor diaries to track motor fluctuations in Parkinson's disease. npj Digit. Med. 3, 6 (2020). https://doi.org/10.1038/s41746-019-0214-x.

Human research participants

Policy information about studies involving human research participants and Sex and Gender in Research.

Reporting on sex and gender	This information was not made accessible to us. The data we received was anonymized and contained no patient-specific demographic details, just sensor data files, MDS-UPDRS scores from clinicians, and ON/OFF state.
Population characteristics	Population characteristics can be found in:<br><br>Erb, M.K., Karlin, D.R., Ho, B.K. et al. mHealth and wearable technology should replace motor diaries to track motor fluctuations in Parkinson's disease. npj Digit. Med. 3, 6 (2020). https://doi.org/10.1038/s41746-019-0214-x.<br><br>This manuscript describes 4 studies that were overseen by 4 separate institutional IRBs. We use data from Study #2 and Study #3. In our manuscript, Study #2 is referred to as Study 1 (conducted at Tufts Medical Center) and Study #3 is referred to as Study 2 (conducted at Spaulding Rehabilitation Hospital). We did not receive demographic information as part of the anonymized data, but the manuscript by Erb et al. contains the population characteristics for all 4 data collection studies they describe.
Recruitment	People with Parkinson's Disease were recruited through IRB approved advertising materials, by word-of-mouth in the clinics of several medical centers across Boston MA, as well as through Parkinson's disease advocacy organizations including services that the Michael J. Fox Foundation maintains to connect people with PD to ongoing clinical research in the field.<br><br>Source: Erb, M.K., Karlin, D.R., Ho, B.K. et al. mHealth and wearable technology should replace motor diaries to track motor fluctuations in Parkinson's disease. npj Digit. Med. 3, 6 (2020). https://doi.org/10.1038/s41746-019-0214-x.
Ethics oversight	Data collection for Study 1 was carried out at the Clinical and Translational Research Center at Tufts Medical Center and all study procedures were approved by the Tufts Health Sciences Campus Institutional Review Board. Study 2 was carried out at Spaulding Rehabilitation Hospital and all procedures were approved by the local Institutional Review Board. Written informed consent was obtained from all participants and all relevant ethical regulations were complied with.<br><br>Source: Erb, M.K., Karlin, D.R., Ho, B.K. et al. mHealth and wearable technology should replace motor diaries to track motor fluctuations in Parkinson's disease. npj Digit. Med. 3, 6 (2020). https://doi.org/10.1038/s41746-019-0214-x.

Note that full information on the approval of the study protocol must also be provided in the manuscript.

Field-specific reporting

Please select the one below that is the best fit for your research. If you are not sure, read the appropriate sections before making your selection.

☒ Life sciences ☐ Behavioural & social sciences ☐ Ecological, evolutionary & environmental sciences

For a reference copy of the document with all sections, see nature.com/documents/nr-reporting-summary-flat.pdf

Life sciences study design

All studies must disclose on these points even when the disclosure is negative.

Sample size	We estimated sample sizes based on an ability to detect a significant change in total MDS-UPDRS Part III subscale score with at least 80% power.<br><br>Source: Erb, M.K., Karlin, D.R., Ho, B.K. et al. mHealth and wearable technology should replace motor diaries to track motor fluctuations in Parkinson's disease. npj Digit. Med. 3, 6 (2020). https://doi.org/10.1038/s41746-019-0214-x.
Data exclusions	A total of 26 subjects were recorded in Study 2, but 3 were omitted due to missing UPDRS clinician scores.
Replication	A fully trained CNN or GAN model (for a specific hyperparameter combination) always produced the same results on the fixed test set of 23 Study 2 subjects, no matter how many times it was re-run.
Randomization	Our development set was 10 subjects that were randomly selected from 35 Study 1 subjects. The same 10 subjects were used for all epochs during training for both CNN and GAN models, for use as a check of convergence.<br><br>All studies described herein were observational in nature. No intervention was introduced that would motivate randomizing participants into particular groups. However, certain aspects of the design of the studies were randomized. In Study 1 conducted at Tufts Medical Center, all participants completed 2 recording sessions under different conditions. To focus analysis on that condition and rule out the possibility of an

order effect on the results, the sequence of visit conditions across participants was randomized.

Source: Erb, M.K., Karlin, D.R., Ho, B.K. et al. mHealth and wearable technology should replace motor diaries to track motor fluctuations in Parkinson's disease. npj Digit. Med. 3, 6 (2020). https://doi.org/10.1038/s41746-019-0214-x.

Blinding	Live clinical raters could not be blinded.However, video raters were asked to be blinded to medication state (i.e. when the assessment was happening relative the to the normal levodopa schedule for that participant).<br><br>Source: Erb, M.K., Karlin, D.R., Ho, B.K. et al. mHealth and wearable technology should replace motor diaries to track motor fluctuations in Parkinson's disease. npj Digit. Med. 3, 6 (2020). https://doi.org/10.1038/s41746-019-0214-x.

Reporting for specific materials, systems and methods

We require information from authors about some types of materials, experimental systems and methods used in many studies. Here, indicate whether each material, system or method listed is relevant to your study. If you are not sure if a list item applies to your research, read the appropriate section before selecting a response.

Materials & experimental systems

n/a	Involved in the study
☒	☐ Antibodies
☒	☐ Eukaryotic cell lines
☒	☐ Palaeontology and archaeology
☒	☐ Animals and other organisms
☒	☐ Clinical data
☒	☐ Dual use research of concern

Methods

n/a	Involved in the study
☒	☐ ChIP-seq
☒	☐ Flow cytometry
☒	☐ MRI-based neuroimaging
